# 2,6-Di­amino-4-chloro­pyrimidinium 4-carb­oxy­butano­ate

**DOI:** 10.1107/S1600536814015220

**Published:** 2014-07-05

**Authors:** Bellarmin Edison, Kasthuri Balasubramani, Kaliyaperumal Thanigaimani, Nuridayanti Che Khalib, Suhana Arshad, Ibrahim Abdul Razak

**Affiliations:** a Department of Chemistry, Government Arts College (Autonomous), Thanthonimalai, Karur 639 005, Tamil Nadu, India; b School of Physics, Universiti Sains Malaysia, 11800 USM, Penang, Malaysia

**Keywords:** crystal structure

## Abstract

In the title mol­ecular salt, C_4_H_6_ClN_4_^+^·C_5_H_7_O_4_^−^, the cation is essentially planar, with a maximum deviation of 0.037 (1) Å for all non-H atoms. The anions are self-assembled through O—H⋯O hydrogen bonds, forming a supra­molecular zigzag chain with graph-set notation *C*(8). In the crystal, the protonated N atom and the 2-amino group of the cation are hydrogen bonded to the carboxyl­ate O atoms of the anion *via* a pair of N—H⋯O hydrogen bonds with an *R*_2_^2^(8) ring motif. This motif further self-organizes through N—H⋯O and O—H⋯O hydrogen bonds, generating an array of six hydrogen bonds, the rings having graph-set notation *R*_3_^2^(8), *R*_2_^2^(8), *R*_4_^2^(8), *R*_2_^2^(8) and *R*_3_^2^(8). In addition, another type of *R*_2_^2^(8) motif is formed by inversion-related pyrimidinium cations *via* N—H⋯N hydrogen bonds, forming a two-dimensional network parallel to (101).

## Related literature   

For applications of pyrimidine derivatives, see: Condon *et al.* (1993 ▶); Maeno *et al.* (1990 ▶); Gilchrist (1997 ▶). For applications of glutaric acid, see: Windholz (1976 ▶). For the conformation of glutaric acid, see: Saraswathi *et al.* (2001 ▶); Stanley *et al.* (2002 ▶). For related structures, see: Thanigaimani *et al.* (2012*a* ▶,*b* ▶); Thanigaimani & Mu­thiah (2010 ▶). For hydrogen-bond motifs, see: Bernstein *et al.* (1995 ▶). For bond-length data, see: Allen *et al.* (1987 ▶).
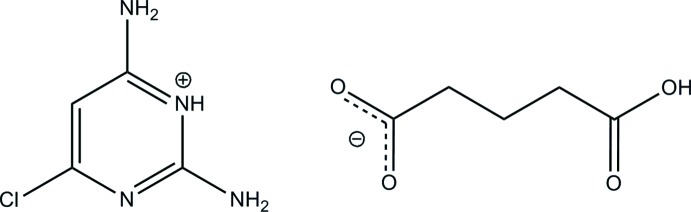


## Experimental   

### 

#### Crystal data  

C_4_H_6_ClN_4_^+^·C_5_H_7_O_4_^−^
*M* *_r_* = 276.68
Monoclinic, 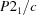

*a* = 5.1582 (1) Å
*b* = 23.2339 (5) Å
*c* = 9.8858 (2) Å
β = 94.7949 (12)°
*V* = 1180.62 (4) Å^3^
*Z* = 4
Mo *K*α radiation
μ = 0.34 mm^−1^
*T* = 296 K
0.54 × 0.24 × 0.21 mm

#### Data collection  

Bruker SMART APEXII DUO CCD area-detector diffractometer
Absorption correction: multi-scan (*SADABS*; Bruker, 2009 ▶) *T*_min_ = 0.838, *T*_max_ = 0.932
31054 measured reflections
3121 independent reflections
2402 reflections with *I* > 2σ(*I*)
*R*_int_ = 0.030

#### Refinement  

*R*[*F* ^2^ > 2σ(*F* ^2^)] = 0.040
*wR*(*F* ^2^) = 0.099
*S* = 1.04
3121 reflections
187 parameters
H atoms treated by a mixture of independent and constrained refinement
Δρ_max_ = 0.24 e Å^−3^
Δρ_min_ = −0.27 e Å^−3^

### 

Data collection: *APEX2* (Bruker, 2009 ▶); cell refinement: *SAINT* (Bruker, 2009 ▶); data reduction: *SAINT*; program(s) used to solve structure: *SHELXTL* (Sheldrick, 2008 ▶); program(s) used to refine structure: *SHELXTL*; molecular graphics: *SHELXTL*; software used to prepare material for publication: *SHELXTL* and *PLATON* (Spek, 2009 ▶).

## Supplementary Material

Crystal structure: contains datablock(s) global, I. DOI: 10.1107/S1600536814015220/sj5418sup1.cif

Structure factors: contains datablock(s) I. DOI: 10.1107/S1600536814015220/sj5418Isup2.hkl

Supporting information file. DOI: 10.1107/S1600536814015220/sj5418Isup3.cml

CCDC reference: 1010934

Additional supporting information:  crystallographic information; 3D view; checkCIF report

## Figures and Tables

**Table 1:** Hydrogen-bond geometry (Å, °)

*D*—H⋯*A*	*D*—H	H⋯*A*	*D*⋯*A*	*D*—H⋯*A*
N4—H1*N*4⋯O2^i^	0.91 (2)	1.99 (2)	2.7950 (19)	147.4 (18)
N2—H2*N*2⋯N3^ii^	0.85 (2)	2.23 (2)	3.079 (2)	176.9 (18)
N2—H1*N*2⋯O4^iii^	0.87 (2)	2.15 (2)	3.0140 (18)	175.5 (18)
N4—H2*N*4⋯O2^iv^	0.87 (2)	1.92 (2)	2.7904 (18)	175 (2)
N1—H1*N*1⋯O1^iv^	0.90 (2)	1.80 (2)	2.6924 (17)	177 (2)
O4—H1*O*4⋯O1^v^	0.94 (3)	1.67 (3)	2.5480 (15)	155 (3)

Symmetry codes: (i) 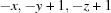; (ii) 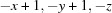; (iii) 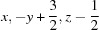; (iv) 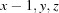; (v) 
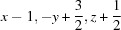
.

**Fig. 1.**
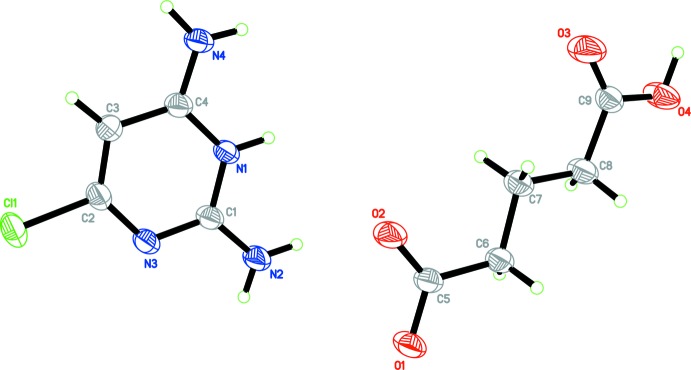
The molecular structure of the title compound with atom labels with 50% probability displacement ellipsoids.

**Fig. 2.**
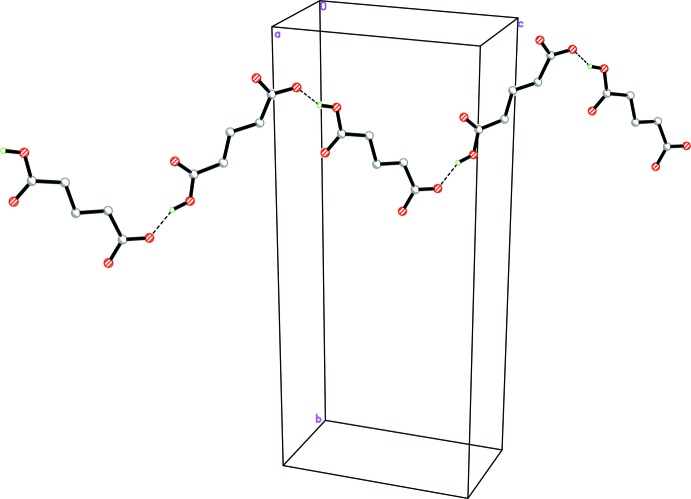
Carboxyl-carboxylate interactions made up of hydrogen glutarate anion

**Fig. 3.**
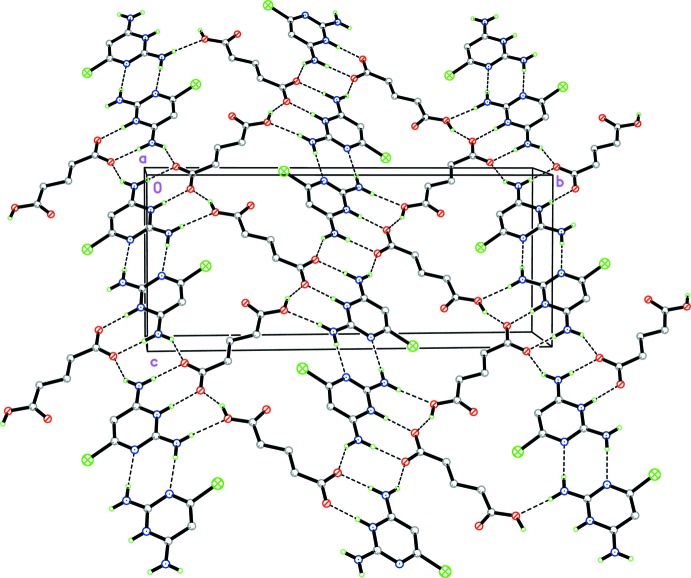
The crystal packing of (I), showing hydrogen-bonded (dashed lines) two-dimensional networks parallel to the bc-plane. The H atoms not involved in the intermolecular interactions have been omitted for clarity.

## supplementary crystallographic information

### S1. Comment

Pyrimidine derivatives are very important molecules in biology and have many application in the areas of pesticide and pharmaceutical agents (Condon *et al.*, 1993). For example, imazosulfuron, ethirmol and mepanipyrim have been commercialized as agrochemicals (Maeno *et al.*, 1990). Pyrimidine derivatives have also been developed as antiviral agents, such as AZT, which is the most widely-used anti-AIDS drug (Gilchrist, 1997). Glutaric acid (pentanedioic acid) is a dicarboxylic acid with five carbon atoms, occurring in plant and animal tissues. Glutaric acid is found in the blood and urine. It is used in the synthesis of phamaceuticals, surfactants and metal finishing compounds. Alpha-ketoglutaic acid is used in dietary supplements to improve protein synthesis (Windholz, 1976). The related crystal structures of Bis(2,6-diamino-4-chloropyrimidin-1-ium) fumarate (Thanigaimani *et al.*, 2012*a*) and 2,6-diamno-4-chloropyrimidine-benzoic acid (1/1) (Thanigaimani *et al.*, 2012*b*) have been recently reported. In order to study some interesting hydrogen bonding interactions, the crystal structure determination of the title compound, (I), was carried out.

The asymmetric unit of the title compound consists of a 2,6-diamino-4-chloropyrimidinium cation and a hydrogen glutarate anion (Fig. 1). The 2,6-diamino-4-chloropyrimidinium cation is essentially planar, with a maximum deviation of 0.016 (1) Å for atom N1. In the 2,6-diamino-4-chloropyrimidinium cation, a wider than normal angle [C1–N1–C6 = 121.44 (12)°] is subtented at the protonated N1 atom. In the hydrogen glutarate anion, C5/C6/C7/C8/C9 plane makes a dihedral angle of 9.67 (12) ° with 2,6-diamino-4-chloropyrimidinium cation. The backbone conformation of the hydrogen glutarate anion can be described by the two torsion angles C5–C6–C7–C8 of -171.89 (13)° and C6–C7–C8–C9 of -176.36 (13)°. As evident from the torsion angles, the hydrogenglutarate anion is in a fully extended conformation (Saraswathi *et al.*, 2001) of the two carboxyl groups, one is deprotonated while the other is not. The bond lengths and angles (Allen *et al.*, 1987) are within normal ranges.

In the crystal packing, the protonated N atom the 2-amino group of the cation are hydrogen bonded to the carboxylate O atoms of the anion *via* a pair of N1—H1N1···O1^iv^ and N4—H2N4···O2^iv^ hydrogen bonds (symmetry code in Table 1), forming *R*_2_^2^(8) (Bernstein *et al.*, 1995) ring motif. This motif further self organizes through N4—H1N4···O2^i^, N2—H1N2···O4^iii^ and O4—H1O4···O1^v^ hydrogen bonds (symmetry code in Table 1), to generate an array of six hydrogen bonds with the rings having the graph-set notations of *R*_3_^2^(8), *R*_2_^2^(8), *R*_4_^2^(8), *R*_2_^2^(8) and *R*_3_^2^(8). The hydrogen glutarate anion self-assemble *via* O4—H1O4···O1 hydrogen bonds to form a one-dimensional supramolecular zigzag infinite chain, with the graph-set notation C(8); this is shown in Fig. 2. This type of head-to-tail fashion of hydrogen glutarate ions has also been observed in the crystal structure of pyrimethamine hydrogen glutarate (Stanley *et al.*, 2002). The inversion-centre-related to the 2,6-diamino-4-chloropyrimidinium cations are also base-paired *via* N2—H2N2···N3^ii^ hydrogen bonds involving the unprotonated pyrimidine N atom and the 2-amino group (symmetry code in Table 1). This type of base pairing, also with an *R*_2_^2^(8) motif, has been observed in many diaminopyrimidiniumcarboxylate salts (Thanigaimani & Muthiah, 2010). These ring motifs extend to give a sheet parallel to (101) plane as shown in Fig 3.

### S2. Experimental

Hot methanol solutions (20 ml) of 2,6-diamino-4-chloropyrimidine (36 mg, Aldrich) and glutaric acid (33 mg, Merck) were mixed and warmed over a heating magnetic stirrer hotplate for a few minutes. The resulting solution was allowed to cool slowly at room temperature and crystals of the title compound (I) appeared after a few days.

### S3. Refinement

O– and N-bound H atoms were located in a difference Fourier maps and allowed to be refined freely [O–H = 0.94 (3) Å and N–H = 0.85 (2)–0.94 (3) Å]. The remaining hydrogen atoms were positioned geometrically [C–H= 0.93–0.97 Å] and were refined using a riding model, with *U*_iso_(H)=1.2 *U*_eq_(C).

### Crystal data

**Table:**

C_4_H_6_ClN_4_^+^·C_5_H_7_O_4_^−^	*F*(000) = 576
*M**_r_* = 276.68	*D*_x_ = 1.557 Mg m^−^^3^
Monoclinic, *P*2_1_/*c*	Mo *K*α radiation, λ = 0.71073 Å
Hall symbol: -P 2ybc	Cell parameters from 9974 reflections
*a* = 5.1582 (1) Å	θ = 2.3–28.9°
*b* = 23.2339 (5) Å	µ = 0.34 mm^−^^1^
*c* = 9.8858 (2) Å	*T* = 296 K
β = 94.7949 (12)°	Block, colourless
*V* = 1180.62 (4) Å^3^	0.54 × 0.24 × 0.21 mm
*Z* = 4	

### Data collection

**Table:**

Bruker SMART APEXII DUO CCD area-detector diffractometer	3121 independent reflections
Radiation source: fine-focus sealed tube	2402 reflections with *I* > 2σ(*I*)
Graphite monochromator	*R*_int_ = 0.030
φ and ω scans	θ_max_ = 29.0°, θ_min_ = 1.8°
Absorption correction: multi-scan (*SADABS*; Bruker, 2009)	*h* = −7→7
*T*_min_ = 0.838, *T*_max_ = 0.932	*k* = −31→31
31054 measured reflections	*l* = −13→13

### Refinement

**Table:**

Refinement on *F*^2^	Primary atom site location: structure-invariant direct methods
Least-squares matrix: full	Secondary atom site location: difference Fourier map
*R*[*F*^2^ > 2σ(*F*^2^)] = 0.040	Hydrogen site location: inferred from neighbouring sites
*wR*(*F*^2^) = 0.099	H atoms treated by a mixture of independent and constrained refinement
*S* = 1.04	*w* = 1/[σ^2^(*F*_o_^2^) + (0.0384*P*)^2^ + 0.4919*P*] where *P* = (*F*_o_^2^ + 2*F*_c_^2^)/3
3121 reflections	(Δ/σ)_max_ < 0.001
187 parameters	Δρ_max_ = 0.24 e Å^−^^3^
0 restraints	Δρ_min_ = −0.27 e Å^−^^3^

### Special details

**Table:**

Geometry. All e.s.d.'s (except the e.s.d. in the dihedral angle between two l.s. planes) are estimated using the full covariance matrix. The cell e.s.d.'s are taken into account individually in the estimation of e.s.d.'s in distances, angles and torsion angles; correlations between e.s.d.'s in cell parameters are only used when they are defined by crystal symmetry. An approximate (isotropic) treatment of cell e.s.d.'s is used for estimating e.s.d.'s involving l.s. planes.
Refinement. Refinement of *F*^2^ against ALL reflections. The weighted *R*-factor *wR* and goodness of fit *S* are based on *F*^2^, conventional *R*-factors *R* are based on *F*, with *F* set to zero for negative *F*^2^. The threshold expression of *F*^2^ > σ(*F*^2^) is used only for calculating *R*-factors(gt) *etc*. and is not relevant to the choice of reflections for refinement. *R*-factors based on *F*^2^ are statistically about twice as large as those based on *F*, and *R*- factors based on ALL data will be even larger.

### Fractional atomic coordinates and isotropic or equivalent isotropic displacement parameters (Å^2^)

**Table:**

	*x*	*y*	*z*	*U*_iso_*/*U*_eq_	
Cl1	0.16574 (11)	0.356710 (18)	0.02821 (5)	0.05343 (16)	
O1	0.9550 (2)	0.61641 (5)	0.37186 (13)	0.0452 (3)	
O2	0.6452 (2)	0.58299 (5)	0.49189 (13)	0.0457 (3)	
O4	0.2839 (3)	0.82514 (5)	0.75083 (13)	0.0456 (3)	
O3	0.1675 (3)	0.73460 (5)	0.78386 (15)	0.0566 (4)	
N1	0.0480 (3)	0.51507 (5)	0.25300 (13)	0.0313 (3)	
N2	0.3649 (3)	0.55724 (6)	0.13291 (16)	0.0410 (4)	
N3	0.2615 (3)	0.46281 (5)	0.08966 (13)	0.0336 (3)	
N4	−0.2630 (3)	0.47569 (6)	0.37842 (15)	0.0388 (3)	
C1	0.2246 (3)	0.51130 (6)	0.15836 (15)	0.0306 (3)	
C2	0.1140 (3)	0.41827 (6)	0.12105 (16)	0.0326 (3)	
C3	−0.0637 (3)	0.41698 (6)	0.21495 (16)	0.0352 (4)	
H3A	−0.1570	0.3839	0.2319	0.042*	
C4	−0.0981 (3)	0.46873 (6)	0.28496 (15)	0.0306 (3)	
C5	0.7825 (3)	0.62305 (6)	0.45526 (16)	0.0317 (3)	
C6	0.7534 (3)	0.68300 (6)	0.51111 (17)	0.0333 (3)	
H6A	0.7280	0.7095	0.4353	0.040*	
H6B	0.9152	0.6935	0.5623	0.040*	
C7	0.5326 (3)	0.69147 (6)	0.60184 (16)	0.0344 (4)	
H7A	0.3721	0.6768	0.5565	0.041*	
H7B	0.5692	0.6700	0.6854	0.041*	
C8	0.5006 (3)	0.75477 (7)	0.63429 (18)	0.0371 (4)	
H8A	0.6670	0.7694	0.6725	0.045*	
H8B	0.4566	0.7752	0.5499	0.045*	
C9	0.2995 (3)	0.76880 (6)	0.73030 (16)	0.0321 (3)	
H1N4	−0.364 (4)	0.4456 (9)	0.3998 (19)	0.050 (6)*	
H2N2	0.469 (4)	0.5531 (8)	0.071 (2)	0.049 (6)*	
H1N2	0.341 (4)	0.5903 (9)	0.171 (2)	0.048 (5)*	
H2N4	−0.282 (4)	0.5094 (9)	0.4155 (19)	0.046 (5)*	
H1N1	0.023 (4)	0.5489 (9)	0.294 (2)	0.052 (6)*	
H1O4	0.153 (6)	0.8363 (12)	0.805 (3)	0.092 (8)*	

### Atomic displacement parameters (Å^2^)

**Table:**

	*U*^11^	*U*^22^	*U*^33^	*U*^12^	*U*^13^	*U*^23^
Cl1	0.0729 (4)	0.0304 (2)	0.0614 (3)	−0.0024 (2)	0.0322 (3)	−0.01516 (19)
O1	0.0534 (8)	0.0268 (6)	0.0612 (8)	−0.0052 (5)	0.0391 (6)	−0.0065 (5)
O2	0.0517 (8)	0.0280 (6)	0.0626 (8)	−0.0071 (5)	0.0359 (6)	−0.0052 (5)
O4	0.0556 (8)	0.0281 (6)	0.0579 (8)	0.0063 (5)	0.0328 (6)	−0.0002 (5)
O3	0.0675 (9)	0.0356 (7)	0.0734 (9)	−0.0098 (6)	0.0450 (8)	−0.0061 (6)
N1	0.0372 (8)	0.0229 (6)	0.0365 (7)	0.0004 (5)	0.0189 (6)	−0.0024 (5)
N2	0.0496 (9)	0.0274 (7)	0.0505 (9)	−0.0047 (6)	0.0301 (7)	−0.0040 (6)
N3	0.0393 (8)	0.0270 (6)	0.0370 (7)	0.0013 (5)	0.0182 (6)	−0.0028 (5)
N4	0.0454 (9)	0.0291 (7)	0.0457 (8)	−0.0021 (6)	0.0270 (7)	−0.0021 (6)
C1	0.0345 (8)	0.0266 (7)	0.0327 (8)	0.0026 (6)	0.0142 (6)	0.0015 (6)
C2	0.0394 (9)	0.0245 (7)	0.0355 (8)	0.0024 (6)	0.0117 (7)	−0.0034 (6)
C3	0.0422 (9)	0.0246 (7)	0.0411 (9)	−0.0040 (6)	0.0177 (7)	−0.0010 (6)
C4	0.0332 (8)	0.0267 (7)	0.0338 (8)	0.0014 (6)	0.0128 (6)	0.0024 (6)
C5	0.0326 (8)	0.0271 (7)	0.0376 (8)	−0.0006 (6)	0.0153 (7)	−0.0017 (6)
C6	0.0340 (8)	0.0262 (7)	0.0418 (8)	−0.0014 (6)	0.0157 (7)	−0.0046 (6)
C7	0.0371 (9)	0.0280 (7)	0.0404 (9)	0.0004 (6)	0.0166 (7)	−0.0033 (6)
C8	0.0418 (9)	0.0286 (7)	0.0439 (9)	−0.0002 (7)	0.0212 (8)	−0.0033 (7)
C9	0.0344 (8)	0.0294 (7)	0.0335 (8)	0.0017 (6)	0.0093 (7)	−0.0028 (6)

### Geometric parameters (Å, º)

**Table:**

Cl1—C2	1.7321 (15)	N4—H1N4	0.91 (2)
O1—C5	1.2720 (17)	N4—H2N4	0.87 (2)
O2—C5	1.2416 (18)	C2—C3	1.358 (2)
O4—C9	1.3281 (18)	C3—C4	1.406 (2)
O4—H1O4	0.94 (3)	C3—H3A	0.9300
O3—C9	1.1982 (19)	C5—C6	1.511 (2)
N1—C1	1.3624 (18)	C6—C7	1.520 (2)
N1—C4	1.3661 (19)	C6—H6A	0.9700
N1—H1N1	0.90 (2)	C6—H6B	0.9700
N2—C1	1.325 (2)	C7—C8	1.517 (2)
N2—H2N2	0.85 (2)	C7—H7A	0.9700
N2—H1N2	0.87 (2)	C7—H7B	0.9700
N3—C2	1.3361 (19)	C8—C9	1.499 (2)
N3—C1	1.3373 (18)	C8—H8A	0.9700
N4—C4	1.3173 (19)	C8—H8B	0.9700
			
C9—O4—H1O4	114.7 (17)	O2—C5—C6	120.54 (13)
C1—N1—C4	121.44 (13)	O1—C5—C6	116.42 (13)
C1—N1—H1N1	119.6 (13)	C5—C6—C7	115.92 (12)
C4—N1—H1N1	118.9 (13)	C5—C6—H6A	108.3
C1—N2—H2N2	115.6 (13)	C7—C6—H6A	108.3
C1—N2—H1N2	121.9 (13)	C5—C6—H6B	108.3
H2N2—N2—H1N2	122.2 (19)	C7—C6—H6B	108.3
C2—N3—C1	115.26 (12)	H6A—C6—H6B	107.4
C4—N4—H1N4	119.0 (12)	C8—C7—C6	110.52 (12)
C4—N4—H2N4	120.4 (13)	C8—C7—H7A	109.5
H1N4—N4—H2N4	120.4 (18)	C6—C7—H7A	109.5
N2—C1—N3	118.62 (13)	C8—C7—H7B	109.5
N2—C1—N1	119.06 (14)	C6—C7—H7B	109.5
N3—C1—N1	122.32 (13)	H7A—C7—H7B	108.1
N3—C2—C3	127.27 (14)	C9—C8—C7	115.99 (13)
N3—C2—Cl1	113.61 (11)	C9—C8—H8A	108.3
C3—C2—Cl1	119.11 (12)	C7—C8—H8A	108.3
C2—C3—C4	115.95 (14)	C9—C8—H8B	108.3
C2—C3—H3A	122.0	C7—C8—H8B	108.3
C4—C3—H3A	122.0	H8A—C8—H8B	107.4
N4—C4—N1	117.81 (14)	O3—C9—O4	122.82 (14)
N4—C4—C3	124.44 (14)	O3—C9—C8	125.75 (14)
N1—C4—C3	117.75 (13)	O4—C9—C8	111.43 (13)
O2—C5—O1	123.03 (14)		
			
C2—N3—C1—N2	179.75 (16)	C1—N1—C4—C3	−1.2 (2)
C2—N3—C1—N1	−0.4 (2)	C2—C3—C4—N4	179.39 (17)
C4—N1—C1—N2	−178.74 (16)	C2—C3—C4—N1	0.0 (2)
C4—N1—C1—N3	1.4 (2)	O2—C5—C6—C7	−5.6 (2)
C1—N3—C2—C3	−0.9 (3)	O1—C5—C6—C7	175.33 (15)
C1—N3—C2—Cl1	179.29 (12)	C5—C6—C7—C8	−171.88 (15)
N3—C2—C3—C4	1.1 (3)	C6—C7—C8—C9	−176.36 (15)
Cl1—C2—C3—C4	−179.10 (13)	C7—C8—C9—O3	1.1 (3)
C1—N1—C4—N4	179.40 (15)	C7—C8—C9—O4	−179.20 (15)

### Hydrogen-bond geometry (Å, º)

**Table:**

*D*—H···*A*	*D*—H	H···*A*	*D*···*A*	*D*—H···*A*
N4—H1*N*4···O2^i^	0.91 (2)	1.99 (2)	2.7950 (19)	147.4 (18)
N2—H2*N*2···N3^ii^	0.85 (2)	2.23 (2)	3.079 (2)	176.9 (18)
N2—H1*N*2···O4^iii^	0.87 (2)	2.15 (2)	3.0140 (18)	175.5 (18)
N4—H2*N*4···O2^iv^	0.87 (2)	1.92 (2)	2.7904 (18)	175 (2)
N1—H1*N*1···O1^iv^	0.90 (2)	1.80 (2)	2.6924 (17)	177 (2)
O4—H1*O*4···O1^v^	0.94 (3)	1.67 (3)	2.5480 (15)	155 (3)

Symmetry codes: (i) −*x*, −*y*+1, −*z*+1; (ii) −*x*+1, −*y*+1, −*z*; (iii) *x*, −*y*+3/2, *z*−1/2; (iv) *x*−1, *y*, *z*; (v) *x*−1, −*y*+3/2, *z*+1/2.
